# Metal-based nano-delivery platform for treating bone disease and regeneration

**DOI:** 10.3389/fchem.2022.955993

**Published:** 2022-08-09

**Authors:** Yanhua Liu, Zhengyi Xu, Mingxin Qiao, He Cai, Zhou Zhu

**Affiliations:** State Key Laboratory of Oral Diseases, National Clinical Research Center for Oral Diseases, West China Hospital of Stomatology, Sichuan University, Chengdu, China

**Keywords:** metal-based nanocarriers, drug deivlery, bone disease, bone regeneration, tissue engineering

## Abstract

Owing to their excellent characteristics, such as large specific surface area, favorable biosafety, and versatile application, nanomaterials have attracted significant attention in biomedical applications. Among them, metal-based nanomaterials containing various metal elements exhibit significant bone tissue regeneration potential, unique antibacterial properties, and advanced drug delivery functions, thus becoming crucial development platforms for bone tissue engineering and drug therapy for orthopedic diseases. Herein, metal-based drug-loaded nanomaterial platforms are classified and introduced, and the achievable drug-loading methods are comprehensively generalized. Furthermore, their applications in bone tissue engineering, osteoarthritis, orthopedic implant infection, bone tumor, and joint lubrication are reviewed in detail. Finally, the merits and demerits of the current metal-based drug-loaded nanomaterial platforms are critically discussed, and the challenges faced to realize their future applications are summarized.

## 1 Introduction

To achieve superior bone repair effects, the development of strategies to treat different bone diseases, such as bone defects, bone infections, fractures, osteoarthritis, osteoporosis, and bone tumors, has become a major public health issue (Chindamo et al., 2020). Bone is the hardest connective tissue in body, and its diseases can lead to restricted movements or even death (Hou et al., 2020). However, there are still many challenges in the diagnosis and treatments of bone diseases. Some bone diseases such as early-stage bone tumors are difficult to be detected by conventional diagnostic methods. It usually requires large doses to reach bone tissue for drugs given by mouth or bolus (Carbone et al., 2017). In addition, the healing cycle of bone tissue is long. Therefore, strict requirements about the release kinetics of therapeutic molecules are necessary for treating bone infection, inflammation or defect. A few emerging technologies, including tissue engineering material transplantation (Liu J. Y. et al., 2021), stem cell technology (Shang et al., 2021), and nanomedicine (Qiao K. et al., 2022), are potential means to promote the treatment of complex bone diseases. In particular, nanomedicine, based on various nanobiomaterials has significantly accelerated the diagnosis research, treatment, and regeneration of bone diseases, and has obtained numerous achievements (Chen et al., 2020; Hagaman et al., 2021). There are a wide variety of nanobiomaterials, including carbon-based, metal-based, virus-based, lipid-based, polymer-based, liposomes, cubes, micelles, exosomes, and cell membrane coatings (Sharma et al., 2021). Nanobiomaterials exhibit numerous excellent properties: nanoscale, large specific surface area, adjustable volume, favorable biocompatibility, and abundant modifiable surfaces (Eivazzadeh-Keihan et al., 2020b; Anastasio et al., 2021). Therefore, they have various applications such as nano-delivery systems (Vangijzegem et al., 2019; Hu S. et al., 2021), biomaterial modification (Liu et al., 2020d; Zhong et al., 2020; Xue Y. et al., 2021), biosensors (Fiorani et al., 2019; Singh et al., 2022), *in vivo* tracking, and imaging agents (Liu Y. et al., 2021; Kalva et al., 2022), gas storage (Wang X. et al., 2021; Zhu et al., 2022), and chemical catalysis (Fang et al., 2021; Khalil et al., 2021). Owing to these excellent properties, nanobiomaterials, particularly nanodelivery platforms, are attractive for the diagnosis and treatment of bone diseases as well as tissue repair. -The characteristic diagnosis and treatment of bone diseases by nanomaterials is particularly attractive. Nanomaterials have large comparative area and surface activity, so can achieve flexible bone-targeting binding, *in vivo* imaging and other functions through physical or chemical modification. Combing nanomaterials with either computed tomography, magnetic resonance imaging (MRI), and/or photoluminescence imaging for specific localization *in vivo* is helpful for the early diagnosis of bone disease (Wang S. et al., 2020). Nanomaterials have excellent loading capacity for bioactive factors, antibacterial agents, antitumor drugs, antibiotic drugs, gene molecules, etc. Required for the treatment of bone diseases (Kumar and Madhumathi, 2016; Li et al., 2019a; Zhao et al., 2020; Halim et al., 2021). More importantly, nanomaterials play a prominent role in reducing drug toxicity, increasing bioavailability, and improving pharmacokinetics and biodistribution, thus providing great potential for new breakthroughs in bone disease treatment (Liu G. et al., 2021; Li et al., 2022).

Compared with organic nanomaterials such as micelles, lipid-based and polymer-based, the metal-based nano-delivery platforms (MNPs) have unique features and advantages due to the inclusion of various metal elements. Considering the important role of metal ions in the metabolism and operation of the human body, particularly the physiological activities of bone tissues, the MNPs have exhibited considerable application potential in bone tissue engineering repair as well as the field of bone disease diagnosis and treatment (Fardjahromi et al., 2022). Metal elements exhibit excellent antibacterial and anti-oxidative stress effects (Makvandi et al., 2020), thereby facilitating synergistic anti-bone infection and osteoarthritis effects to the delivered components (Luo et al., 2021); however, a few other metal elements can also endow nano-delivery systems with special targeted delivery (Niculescu and Grumezescu, 2022), photodynamic therapy (PDT) (He et al., 2022), thermodynamic therapy (Boroushaki et al., 2022), bioimaging (Zhong et al., 2021), and stimuli-responsive (Hu et al., 2022) functions, which are more conducive to the efficient diagnosis and treatment of bone diseases or promote bone regeneration, and decrease the simple systemic side effects of drug use. A few of researchers reported that metal nanoparticles (NPs) can be used as nano-antibiotics because of their favorable antibacterial activities and significant potential to combat antibiotic resistance (Cheng et al., 2022). In addition, studies have demonstrated that metal-based nanomaterials possess excellent mechanical properties as well as the intrinsic ability to significantly promote osseointegration, osteoconductivity, and osteoinduction, which has become crucial factors for bone regeneration (Sobolev et al., 2019; Eivazzadeh-Keihan et al., 2020a). Moreover, MNPs can help achieve the controlled release of drugs or active molecules through various stimuli responses, including pH-triggered release systems (Duan et al., 2021), redox reactions (Duan et al., 2018), external thermal effects (Khodaei et al., 2022), magnetic field control (Dehghani et al., 2020), and ultrasonic dynamic stimulation (Li S. et al., 2020), or by the external activation of optical/mechanical stimuli (Feng et al., 2019).

In recent years, increasingly advanced methods have been attempted to use MNPs for the maintenance of bone tissue health. Komal et al. (Rao et al., 2018) placed Hesperidin into gum acacia-stabilized green silver (Ag)NPs for combating rheumatoid arthritis and achieved favorable results. Additional to loading an active ingredient, the co-encapsulation and simultaneous or sequential release of two or more active ingredients can be achieved using MNPs. For example, Yan (Jiang et al., 2021) used the Zn-based zeolitic imidazole framework (ZIF-8) as a carrier to deliver bone morphogenetic protein 2 (BMP-2) and cisplatin, thus defining different spatial distributions and environment-adaptive release patterns of osteogenic growth factors and anti-cancer drugs. In addition, metal-based nanomaterials can serve as bridges for integrating diagnostic and therapeutic components into a single platform. In a study (Wang et al., 2016b), the authors developed a novel core–shell PB@MIL-100(Fe) metal-organic frameworks (d-MOFs) NPs, which could serve as a contrast agent for MRI. Then the (d-MOFs) NPs was reported acting as an imaging agent for fluorescence optics as well as for achieving targeted tumor therapy in pH-responsive manner, and finally synergistically play the role of photothermal and chemotherapy (CT) for ablating tumors in mice. However, although the MNPs have been demonstrated to have significant potential in bone tissue engineering and related disease diagnosis and treatment, numerous questions regarding MNP have been posed. Titanium dioxide NPs have been reported to induce potential cytotoxicity (oxidative stress), genotoxicity, and immunotoxicity (Di Giampaolo et al., 2021). A few scholars have also proposed that the drug loading capacity of MNPs is predominantly unsatisfactory, and the high production costs limit the clinical translation process (Liu et al., 2020c). For oral administration, penetrating the mucus gel layer barrier to the absorption membrane is a significant test for MNPs to exert their efficacy, and MNPs within the 10–150 nm size range are considered to be the most favorable choice for enhancing permeability and exerting their effects (Murugan et al., 2015). Currently, only a few nanomedicines, such as Abraxane^®^ (albumin-bound paclitaxel NPs) and Doxil (a PEGylated liposomal doxorubicin (DOX) formulation), have been approved for clinical use by the US Food and Drug Administration (FDA) (Barenholz, 2012).

**FIGURE 1:**
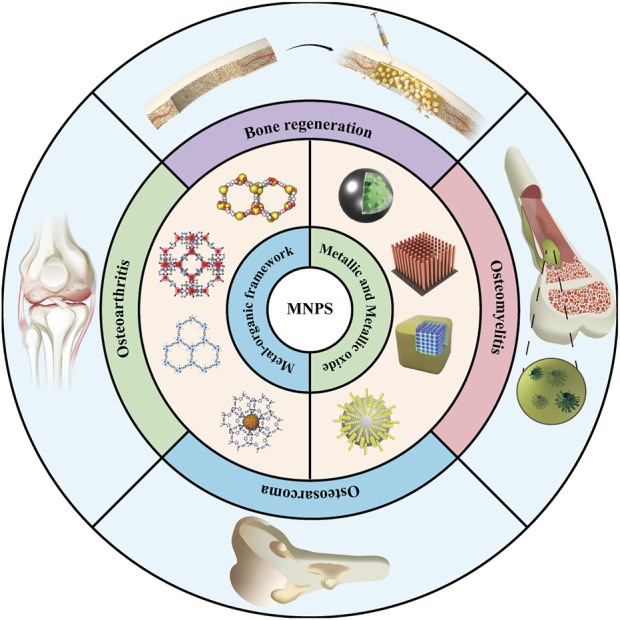
Application of metal-based nano-delivery platform for treating bone disease and regeneration.

Herein, to comprehensively evaluate the status of MNP in the bone health maintenance field and interpret the advantages and disadvantages of such biomaterials for the diagnosis and treatment of bone diseases as well as tissue repair, it is urgently required to summarize the applications status of MNPs targeting bone tissues (Figure 1). This review summarizes the following four aspects: a complete classification and description of the MNPs; then, the method of the MNPs loading active factors is summarized; the application of MNPs to bone-related diseases is further introduced. Specific scenarios are discussed in the third section; the limitations of MNPs at this stage are critically discussed, and it is believed that this review can provide a reference for the application of MNPs in bone tissue-related research in the near future.

## 2 Classification of MNPs

### 2.1 MOFs nano-delivery platform for bone disease and regeneration

**FIGURE 2:**
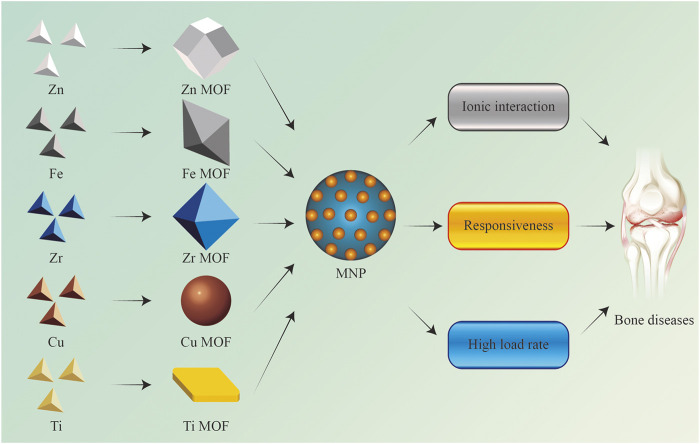
Important components of MNPs: classification and advantages of MOFs.

MOF nanomaterials are a class of crystalline microporous materials that include endless lattices constituting metal ions or clusters and organic ligands connected via strong coordinate bonds (Fang et al., 2018). Compared with other non-metallic nanocarriers such as dendrimers, and mesoporous silica nanoparticles, MOFs have distinct and unique characteristics. First, different from the rigid structure of mesoporous silica nanoparticles, MOFs can flexibly adjust their structures by changing inorganic clusters and organic ligands (length, volume, bond angle, and chirality). Meanwhile, the pores of dendrimers and mesoporous silica nanoparticles are hydrophobic, while the pores of MOFs are amphiphilic (Sharma et al., 2019; Fernandes et al., 2021). All these features can ensure that MOFs can be more efficiently carried for different therapeutic molecules or drugs. Although the metal-free polymeric or liposomal nanocarriers have better stability and biocompatibility (Gupta et al., 2021; Liang et al., 2022). Inorganic metals such as Zn, Ti, Fe, Mg, Cu, Zr, and Co. contained by MOFs are not only essential elements for body, but also participating in important life activities such as cell proliferation, differentiation, and metabolism. More importantly, these inorganic metal elements can also endow MOFs with many other attractive functions, such as antibacterial properties, ionic interaction, magnetism, photothermal response, pH response, chemical catalysis, and enzymatic reactivity. The prevalent MOFs in the treatment of bone disease are classified by metal elements and discussed in the following (Figure 2).

#### 2.1.1 Zn MOFs nano-delivery platform

Nano Zn MOFs comprises Zn (II) covalently linked with 1,4-bis(1H-pyrazol-4yL)-2-X-benzene (H2BDP_X; X = H, NO_2_, NH_2_, OH) (He et al., 2021) and possess various pores sizes in the range of 3.8–28.8 Ǻ. A major subfamily of Zn nano MOFs is the zeolitic imidazolate framework (ZIF), in which Zn (II) ions are connected via imidazolate or imidazole derivatives as organic ligands (Fardjahromi et al., 2022). Since Jiang et al. (Jiang et al., 2009) used an MOF as the Au nanoparticle carrier for the first time, Zn-based MOFs have been widely explored as a nano-delivery platform, and ZIF-8 application is the most representative. The ZIF-8 nano-platform for delivery of biologically active factors or drugs has been used in bone-related diseases or bone tissue engineering regeneration. ZIF-8 is a space-filling packing with the topology of truncated octahedrons and a pore size of 11.6 Ǻ (He et al., 2021). It has been reported that ZIF-8 can effectively deliver one or more small-molecule drugs such as alendronate (Ald), DOX (Xue X. H. et al., 2021), curcumin (Wang Y. T. et al., 2020), risedronate (Cheng et al., 2020), dexamethasone (Ran et al., 2018), 5-fluorouracil, and indocyanine green (ICG) (Ting et al., 2022), and simvastatin (Qiao M. et al., 2022). ZIF-8 has also been demonstrated to be an effective non-viral vector for delivering small noncoding RNAs such as microRNAs (miRNAs) in addition to viral vectors (Feng et al., 2022). Nano ZIF-8 can also be used to simultaneously transport drugs and proteins together; for example, Jiang et al. (Jiang et al., 2021) successfully loaded two substances, BMP-2 and cisplatin, within ZIF-8 to build MNPs for bone regeneration. Furthermore, drugs and genes could be co-delivered by Zn-based MOFs, and Rabiee et al. (Rabiee et al., 2021) had used MOF-5 to simultaneously carry DOX and pCRISPR. Therefore, nano-Zn MOFs have broad prospects for delivering active ingredients, such as a variety of drugs, genes as well as proteins, and can be used as a powerful reserve to accelerate the treatment of bone tissue diseases and bone regeneration.

#### 2.1.2 Fe MOFs nano-delivery platform

Fe-based MOFs is an important constituent member of the Materials of Institute Lavoisier (MIL)-n family (Zhong et al., 2021). Common Fe-based MOFs are MIL -53(Fe), MIL-88A (Fe), MIL-88B(Fe), MIL-100(Fe), and MIL-101(Fe), which are composed of Fe centers and a terephthalate-based linker (1,4-dicarboxylic acid) connected by six oxygen atoms (Al Haydar et al., 2017). Fe-based MIL has been widely studied in biosensing, bioimaging, antibacterial, and drug delivery owing to its excellent properties such as low toxicity, biodegradability, biocompatibility, large pore volume, and surface area with high drug loading capability (Zhong et al., 2021). Fe-based MIL has significant potential as a nano-delivery platform. In particular, except delivering small molecule drugs, such as methotrexate (Ahmadijokani et al., 2021), protocatecholic acid (Xiong et al., 2020), curcumin (Faaizatunnisa et al., 2022), Ald (Golmohamadpour et al., 2018), flurbiprofen (Al Haydar et al., 2017), artemisinin (Wang et al., 2016b), acetaminophen (Pattappan et al., 2022), progesterone, and stavudine (Gordon et al., 2015), MIL family can serve as carrier for the sustained release of small molecule active metabolites, such as WR-1065 (active metabolite of amifostine), and peptides, such as glutathione (Cao et al., 2020). Therefore, Fe MOFs are also functionally rich nano-delivery platforms which can be used to overcome the challenges of bone regeneration and repair as well as bone disease diagnosis and treatment.

#### 2.1.3 Zr MOFs nano-delivery platform

Zr MOFs are considered as promising MOFs for bone tissue engineering and bone disease-related applications owing to their low toxicity as well as high mechanical, thermal, acidic, and aqueous stability (Lazaro et al., 2017). In 2008, Cavka discovered the first Zr MOF, namely Universitetet i Oslo (Uio-66) (Cavka et al., 2008), which comprises inorganic Zr metal and organic ligand 1,4-benzene dicarboxylic acid. Additional to UiO-66, MOF-525, MOF-545 (also known as PCN-222), PCN-221, PCN-223, PCN-224, PCN-225, and NU-902 are commonly used Zr-based MOFs (Yu et al., 2021). The size, porosity, and release behavior of Zr MOFs can be regulated via the synthesis of different functional groups such as -NH_2_ and NO_2_ (Li Z. et al., 2019). Zr MOFs can be used to deliver different drugs or active ingredients when treating different bone diseases. For example, small molecule drugs can be delivered using UiO-66-NH2 NPs such as DOX, cisplatin, temozolomide, and curcumin (Ma et al., 2020; Fytory et al., 2021; Wan et al., 2021; Hu et al., 2022). In addition, a few single metal atoms (Pt, Au, Cu, Ru) can be delivered using PCN-222 (Yu et al., 2022). Moreover, it has been reported that a few immunostimulatory oligonucleotides, such as cytosine–phosphate–guanosine, can be successfully delivered using UiO-66 (Pang et al., 2020). Therefore, owing to their excellent delivery ability, the Zr MOFs nanoplatform is a new candidate as tissue engineering materials for diagnosing and treating bone tissue diseases.

#### 2.1.4 Cu MOFs nano-delivery platform

In recent years, nano Cu MOFs have been frequently explored in the field of tissue engineering and nano-drug loading (Telgerd et al., 2019; Wang T.-L. et al., 2021; Binaeian et al., 2022). Benzene-1,3,5tricarboxylate linkers and Cu ions are connected to form Hong Kong University of Science and Technology-1 (HKUST-1), which is a widely used nano-Cu MOF (Lin et al., 2012). Owing to their numerous advantages, Cu MOFs are suitable candidates for the development of nano-delivery platforms: I) they provide a unique broad-spectrum antibacterial effect (Lee et al., 2021; Wang Z. et al., 2022); II) they possess the ability to stimulate endothelial cell proliferation and differentiation, thereby promoting angiogenesis by simulating hypoxia (Dang et al., 2020); III) they possess an open skeleton framework and excellent chemical stability (Liu et al., 2019); IV) they possess a unique near-infrared (NIR) absorption ability, which can be used to develop photo-thermal therapy (PTT) nano-drug system (Weng et al., 2020; Wang L. et al., 2021; Geng et al., 2022); V) they have a favorable loading capacity and are a good choice for developing chemodynamic therapy (CDT) nanocarriers (Hao et al., 2021b). Recently, small-molecule drugs, such as chlorhexidine (Soltani and Akhbari, 2022), DOX (Gharehdaghi et al., 2021), 5-fluorouracil (Liu W. et al., 2020), methotrexate (Nezhad-Mokhtari et al., 2019), diclofenac sodium, chlorpromazine hydrochloride, amodiaquin dihydrochloride (Liu et al., 2019), and ibuprofen (Javanbakht et al., 2019), have been successfully loaded into nano-drug delivery systems through Cu MOFs. Enzymatically active molecules, such as horseradish peroxidase and glucose oxidase, can also be effectively loaded by Cu MOFs and exhibit superior stabilities than those in the free state (Hao et al., 2021a; Lin et al., 2021). Therefore, nano-Cu MOFs are the widely explored nano-drug delivery platform in recent years.

#### 2.1.5 Ti MOFs nano-delivery platform

Owing to the superior biocompatibility and photocatalytic performance of Ti compared with other metals, Ti MOFs exhibit significant potential for bone tissue engineering and nano-drug loading. In 2009, Dan-Hardi et al. (Dan-Hardi et al., 2009) first reported MIL-125(Ti), which comprises Ti octahedra and terephthalate dianions with accessible pore diameters of 6.13 and 12.55 Å. MIL-125(Ti) has attracted considerable attention in nano-drug loading, and it has been demonstrated to solely load small molecule drugs, such as DOX (Rengaraj et al., 2017), aspirin (Rojas et al., 2019), and ibuprofen (Xie et al., 2018), Ag NPs (Arenas-Vivo et al., 2019) as well as carbon monoxide gas (Jin et al., 2018). Thus, Ti MOFs are also a favorable choice for nano-delivery platform materials.

#### 2.1.6 Other metal MOFs nano-delivery platforms

In addition to the several MOFs mentioned above, other MOF nano-delivery platforms, such as Mg-, Co-, and Ca-based MOFs have potential applications in the bone tissue engineering field. Although further research is required to expand the contents of these MOFs on nano-drug delivery, these MOFs have been attracting increasing research attention in recent years. Mg MOFs have been demonstrated to effectively load small molecule drugs such as icariin (Wang W. et al., 2022), ibuprofen, curcumin (Lawson et al., 2022), alpha-cyano-4-hydroxycinnamate (Hu J. Q. et al., 2021), and IL4 cytokines (Zheng et al., 2020). Additionally, Co. MOFs were used to load drugs, enzymes, and bioactive molecules, such as dimethyloxalylglycine (DMOG) (Li J. K. et al., 2020), olsalazine (Levine et al., 2016), glucose oxidase (Fang et al., 2020), and 4-chloro-N-cyclohexyl-N-(phenylmethyl)-benzamide (Sun et al., 2022). Among the metal elements predominantly present in bone, Ca is also observed to build MOFs serving as nanocarriers for loading drugs such as 5-fluorouracil (Li D. et al., 2020), ibuprofen, and guaiacol (Wei et al., 2017). Overall, further analysis and studies of Mg, Co., and Ca MOFs are required because of their significant potential applications in nano-delivery platforms to overcome bone-related problems.

### 2.2 Metallic and metallic oxide nano-delivery platform

In recent years, metallic and metallic oxide nanoparticles have attracted significant attention. Although non-metallic NPs such as ceramic nanoparticles and polymeric nanoparticles can load with many therapeutic drugs, growth factors or genetic materials, metallic and metallic oxide nanoparticles are still irreplaceable for bone disease treatment. As we all known, metallic and metallic oxide NPs have unique advantages in the prevention or treatment of some infectious bone diseases because not only their good drug delivery ability, but also the excellent antibacterial properties. Not exactly the same as the antibacterial mechanism of metal ions from metal inorganic salts, in fact, the antibacterial properties of metallic and metallic oxide NPs are not only owing to the metal ions they release. Nanoscale size, morphology, and mediated generation of reactive oxide species (ROS) are all important factors for the antibacterial mechanism of this class of MNPs (Godoy-Gallardo et al., 2021). Besides, metallic and metallic oxide NPs have numerous advantages unlike traditional nanomaterials: excellent physical and chemical properties, osseointegration ability, cell labeling and imaging, photothermal response as well as magnetic actuation. Currently, the metallic and metallic oxides being used in nano-delivery platforms are also classified by metal elements and discussed in the following sections (Table 1).

**TABLE 1:** Classification of Metallic and Metallic oxide.

Formula	Cargo loading	Functions	Citations
**Ag**			
Ag/Ag_2_O	Dox	Biochemical sensing	Zeng et al. (2018)
	Protocatechuic acid	Chemical reducibility	Oh et al. (2016)
	Gallic acid	Targeted delivery and reduced side effects	Muhammad et al. (2016)
	Methotrexate	Self-disinfection property	Raafat et al. (2018)
	Propranolol Hcl	Extend release time	Gilevskaya et al. (2018)
	Imatinib	Antimicrobial activity	Kodoth et al. (2019
	Donepezil	Antiviral activity	Li et al. (2016)
	Aman Camptothecin tadine	Anti-cancer activity and inhibitory effect on drug resistance related proteins	Zhan et al. (2017)
	Zanamivir	Reverse influenza virus resistance	Lin et al. (2017)
**Au**			
Au	Alpha-Tocopheryl Succinat,	Targeted chemotherapy and computed tomography imaging of cancer cells	Zhu et al. (2015)
	Ginsenoside Rg3	Magnetic and optical properties and the response to x-ray radiation	Zhang et al. (2020)
	Vg16krkp peptide	Bacteriolytic activity	Chowdhury et al. (2017)
	Chlorin E6	Tumor suppression	Chuang et al. (2020)
	Gemcitabine	Electrostatic interactions	Wang et al. (2018b)
	Methotrexate	Improve its solubility, stability and biodistribution	Bessar et al. (2016)
	Insulin molecules	Targeted delivery	Betzer et al. (2019)
	Gentamicin sulfate	Amplify antibacterial activity	Zou et al. (2020b)
**Cu**			
Cu	DOX	PH-responsive and real-time cell imaging	Li et al. (2021)
	Curcumin	Efficient antimicrobial enzyme carrier	Nor et al. (2021)
	Lysozyme	Antibacterial activity	Targhi et al. (2021)
	Rifampicin	Antimicrobial properties	Wozniak-Budych et al. (2017)
**Zn**			
ZnO	Hesperidin	Antiviral activity	Attia et al. (2021)
	Gentamicin	Antibacterial and antibiofilm	Hemmati et al. (2020)
	Daunorubicin	Carrier for various anti-cancerous drugs	Kumar and Pal (2019)
	Docosahexaenoic acid	Carrying active molecules	Hussein et al. (2019)
	Curcumin	PH-responsive	Kundu et al. (2019)
**Ti**			
TiO_2_	DOX	Enhancing the anticancer efficacy	Chen et al. (2011)
	Gentamicin	Enhancing cell attachment, proliferation, and differentiation	Escobar et al. (2019)
	W	Sonodynamic, chemodynamic, and GSH-depleting activities	Geng et al. (2021)
	Strontium	Osteogenic activity	Han et al. (2021)
	Methylthioadenosine nucleosidase inhibitor	Reduce infection and promote osteogenesis	Li et al. (2020e)
**Fe**			
SIONs/Fe_2_O_3_	Docetaxel	Tumor specific targeting	Nagesh et al. (2016)
	Rifampicin and Tetracycline hydrochloride	Target the specific site to deliver the drug	Prakash et al. (2019)
	DOX, cisplatin, artemisinin and paclitaxel	The wide amount of drug nanocarriers magnetic and biological properties targeting abilities	Vangijzegem et al. (2019)
	Amino-terminal fragment peptide	Target specificity and in vivo imaging	Yang et al. (2009)
	Anti-HER2/neu peptide	Tumour targeting	Mu et al. (2015)
	Anti-CD44 antibody	Magnetomechanical and photothermal treatments	Alsharif et al. (2020)
	Prostate specific membrane antigen	PET-CT scan	Bandini et al. (2017)
	‐cyclodextrin	Super paramagnetic behavior	Sudha et al. (2016)
	Pluronic F127 curcumin	Chemo-hyperthermia	Khodaei et al. (2022)
**Pd**			
Pd	DOX	Effective drug delivery	Shanthi et al. (2015)

#### 2.2.1 Ag and Ag oxide nano-delivery platforms

Owing to the powerful bactericidal function, anti-inflammatory effect, mechanical strength, osteoinductive properties, and enhancement of cell proliferation rate (Shuai et al., 2018), Ag and Ag oxide (Ag_2_O) NPs are promising candidate materials for bone tissue engineering material coatings (Khan et al., 2020; Nardo et al., 2021), scaffold fillers (Xu et al., 2016), bactericides (Cao et al., 2018), and nano-delivery platforms (Raafat et al., 2018). The particle sizes, morphologies, surface chemistries, aggregation levels, and doses of Ag and Ag_2_O NPs affect the cellular response when used as carriers. Ag and Ag_2_O NPs can be used as delivery platforms for various drugs. For example, Zeng et al. used nanographene oxide (NGO)-coated Ag NPs (Ag@NGO) as a nanocarrier to deliver DOX (Zeng et al., 2018). Protocatechuic acid, gallic acid (Oh et al., 2016), methotrexate (Muhammad et al., 2016), propranolol HCl (Raafat et al., 2018), imatinib (Gilevskaya et al., 2018), donepezil (Kodoth et al., 2019), amantadine (Li et al., 2016), camptothecin (Zhan et al., 2017), zanamivir (Lin et al., 2017), and other drugs have been successfully delivered using Ag NPs.

#### 2.2.2 Au nano-delivery platform

Au NPs with excellent biocompatibility, antibacterial ability, physicochemical properties, osseointegration ability, and abundant modifiable surfaces have wide ranging applications such as biocoatings, antimicrobials against drug resistance, and targeted drug carriers (Lee et al., 2018). Au NPs are known to be excellent nano-delivery vehicles for various drug molecules, peptides, proteins, plasmid deoxynucleic acid (pDNA), small interfering ribonucleic acid (siRNA), and chemotherapeutic agents (Li et al., 2015; Mallakpour et al., 2021). Au NPs have attracted considerable attention because of their excellent properties. Numerous teams have successfully achieved the delivery of antioxidant alpha-tocopheryl succinate (α-TOS) (Zhu et al., 2015), ginsenoside Rg3 (Zhang et al., 2020), VG16KRKP antimicrobial peptide (Chowdhury et al., 2017), chlorin e6 (Chuang et al., 2020), gemcitabine (Wang Z. et al., 2018), methotrexate (Bessar et al., 2016), insulin molecules (Betzer et al., 2019), gentamicin sulfate (Zou Y. et al., 2020), as well as various active molecules or drugs, such as gentamicin and ampicillin, demonstrating that Au NPs can be used as a reliable nano-delivery platform.

#### 2.2.3 Cu nano-delivery platform

Compared to Au and Ag, Cu is an inexpensive and readily available metal and among the essential trace elements present in most living organisms. Simultaneously, Cu has gradually become a research hotspot owing to its excellent physical, chemical, electrical and optical properties as well as favorable antibacterial properties. The stable interactions between Cu NPs and various drugs (DOX, curcumin, lysozyme, rifampicin) can form nano-delivery systems with superior performance (Wozniak-Budych et al., 2017; Li et al., 2021; Nor et al., 2021; Targhi et al., 2021).

#### 2.2.4 Zn oxide nano-delivery platform

Zn oxide (ZnO) exists in various forms, such as ZnO nanospheres, nanosheets, nanorods, and nanowires (Carofiglio et al., 2020; Wojnarowicz et al., 2020). Zn ions have been confirmed to effectively promote the mineralization of the extracellular matrix of bone marrow mesenchymal stem cells (BMSCs) (Wang Q. et al., 2018), implant osseointegration (Chen et al., 2017), migration and tube formation of vascular endothelial cells (Nakamura et al., 2020), and antibacterial effects (Yang et al., 2020). ZnO is a high-profile candidate material for bone tissue engineering material modification, bone regeneration, angiogenesis, as well as antibacterial and anti-infection effects. In addition, ZnO has broad prospects as nanocarriers. The loading of ZnO for various drugs can be realized using simple and quick methods. Currently, researchers have successfully used ZnO to load hesperidin (Attia et al., 2021), gentamicin (Hemmati et al., 2020), daunorubicin (Kumar and Pal, 2019), docosahexaenoic acid (Hussein et al., 2019), curcumin (Kundu et al., 2019), and other drugs.

#### 2.2.5 Ti dioxide nano-delivery platform

It is well known that the Ti metal has excellent biocompatibility and osseointegration ability. Ti or Ti alloys have been widely used as implant materials in implants, bone substitute materials, fracture fixation screws, meshes, etc. Based on the excellent properties of Ti, Ti dioxide (TiO_2_) nanomaterials have attracted the attention of researchers. Numerous nanostructures of TiO_2_, such as TiO_2_ nanotubes, NPs, matrices, rods, and whiskers, have emerging applications as delivery platforms for different drugs or active factors. For example, TiO_2_ nanomaterials can deliver multiple small-molecule drugs, such as dexamethasone, temozolomide, curcumin, cisplatin, gambogic acid, valproic acid, DOX, and daunorubicin, as well as effectively deliver methylthioadenosine nucleosidase inhibitor, biologically active protein human recombinant BMP-2 and metal tungsten, strontium, etc. (Chen et al., 2011; Escobar et al., 2019; Li et al., 2020c; Geng et al., 2021; Han et al., 2021). Evidently, TiO_2_ is a powerful nano-delivery platform and an extremely promising material for bone health maintenance.

#### 2.2.6 Iron oxide nano-delivery platform

Superparamagnetic iron oxide NPs (SIONs) are multifunctional nanomaterials of significance in bone disease diagnosis, targeted delivery, and bone replacement material modification because of their unique magnetic and biological properties. Iron oxide NPs have been approved for clinical applications. Magnetic iron oxide NPs have excellent advantages as nanocarriers, because they can efficiently load various drugs and also be imaged or reach target sites *in vivo* via an external magnetic force, enhancing the bioavailability of therapeutic compounds. Studies have demonstrated that SIONs can effectively deliver cisplatin, curcumin, lauric acid, mitoxantrone, artemisinin, docetaxel, paclitaxel, dopamine, 10-hydroxycamptothecin, docetaxel, DOX, rifampicin, tetracycline hydrochloride, and other drugs (Nagesh et al., 2016; Prakash et al., 2019; Vangijzegem et al., 2019). In addition, a few peptides, antibodies, or organic small molecules, such as amino-terminal fragment peptide (Yang et al., 2009), anti-HER2/neu peptide (Mu et al., 2015), anti-CD44 antibody (Alsharif et al., 2020), prostate specific membrane antigen (Bandini et al., 2017), β-cyclodextrin (Sudha et al., 2016), and pluronic F127 polymer (Khodaei et al., 2022)were conjugated on the SION surfaces, consequently enhancing the targeting function of the SIONs delivery system, increasing the specificity and therapeutic effect.

#### 2.2.7 Palladium nano-delivery platform

Palladium is a precious metal material with high mechanical strength, high porosity, as well as anti-microbial, anti-oxidation, and anti-cancer properties. It is a worthy target for various applications including medical biomaterials, nano-delivery carriers, and bone disease treatment. Palladium is an effective nano-delivery platform, which has been demonstrated in several studies, and drugs, such as DOX, has been successfully loaded using palladium NPs (Shanthi et al., 2015).

## 3 Loading method of MNPs

**FIGURE 3:**
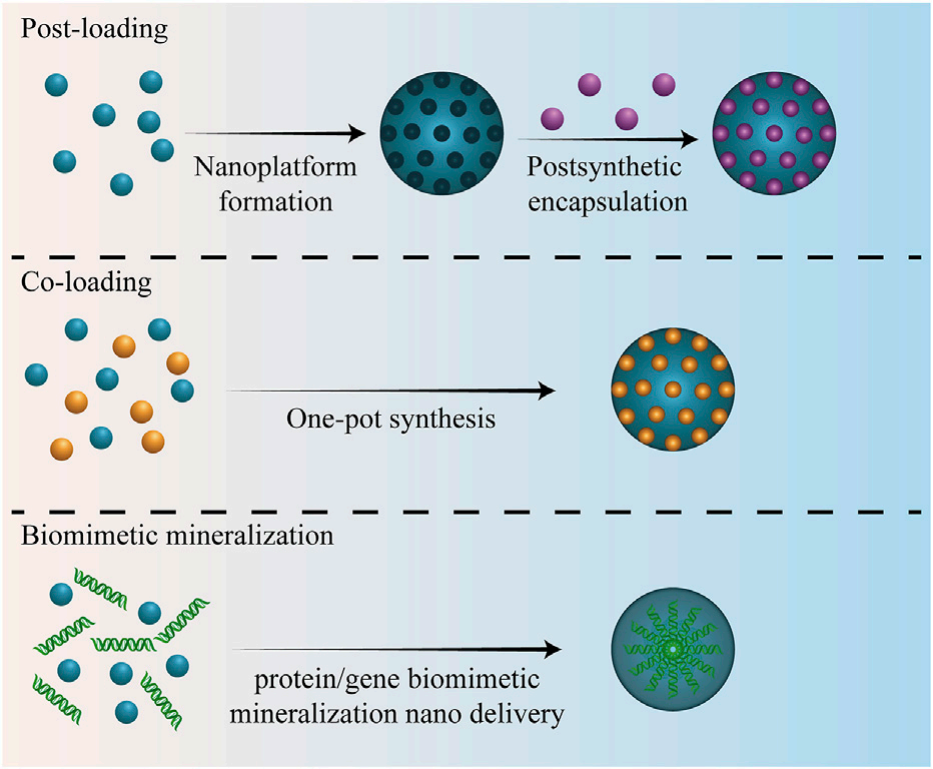
Different loading methods of MNPs.

The MNP loading method affects the loading efficiency and release rule of therapeutic molecules, further affecting the diagnosis and treatment of bone-related diseases or bone regeneration. There are three commonly used methods for MNP loading with therapeutic molecules: post-loading, Co-loading, and biomimetic mineralization (Liu et al., 2020c; Lawson et al., 2021). Different loading methods are suitable for different kinds of MNPs. The three loading methods of MNPs are elaborated in the following subsections (Figure 3).

### 3.1 Post-loading

Post-loading can also be referred to as “postsynthetic encapsulation/loading,” which is a strategy of first fabricating nanocarriers and then loading drugs to achieve high drug loading nano drug-loading systems. Using this method, non-porous nanomaterials can be combined with therapeutic molecules via electrostatic attraction, non-covalent hydrophobic interactions, π–π stacking, and hydrogen bonding. Benefiting from tunable pore, high specific surface areas, and active surfaces, some porous NPs can also be loaded using drugs via this method (Chen et al., 2021). Therefore, postsynthetic encapsulation/loading is a suitable method for most MNPs; however, the difference is that for MNPs with pores, small drug molecules can be encapsulated inside the MNPs through the pore size; for MNPs without sufficient pore sizes or if the pores are already occupied, drug molecules can only be loaded onto the nanomaterial surface or functionalized modified surfaces. This post-loading method is particularly suitable for loading additional therapeutic molecules into MOFs that have already encapsulated the drug. The MNP pore size, drug-to-pore size ratio, and strength of interactions, such as coordination bonds, hydrophobic interactions, electrostatic attraction, and π–π stacking, between the drug and MNPs can all affect the loading efficiency of post-loading (Cao et al., 2020; Lawson et al., 2021). Farhad et al. (Ahmadijokani et al., 2021) post-loaded methotrexate with three MOFs, MIL-53, NH2-MIL-53 and NH2-MIL-101, and observed polar amine groups, larger surface area and pore volume, high positive zeta potential, and NH2-MIL-101 exhibited the highest loading capacity, 457.69 mg/g, of the drug. They proposed that electrostatic interactions, π–π stacking interactions, and H-bonding are the primary mechanisms by which methotrexate is successfully post-loaded. Hu et al. (Hu J. Q. et al., 2021) loaded CHC on Mg-MOF-74 using the “post-loading” method and achieved a loading of 625 mg g^−1^. Post-loading metal NPs can also be used to achieve high drug loading. A few researchers used Polyethylene glycol–poly (ethylene imine) -functionalized Au NPs to load chlorin e6, and the loading content reached 46.4 ± 0.4% (Chuang et al., 2020).

### 3.2 Co-loading

Co-loading, also known as “one-pot synthesis”, refers to the strategy of loading or encapsulating drugs during MNP formation. This loading method is primarily applicable to MOFs, and the therapeutic molecules can be used as building blocks to contribute to MOF formation along with metal ions. The size of the therapeutic molecule affects physical properties, such as the size and charge of the final MOF drug-carrying system, to an extent. One-pot synthesis is simple, convenient, and can facilitate the uniform distribution of drug molecules in the whole MOFs as well as effectively prevent the rapid release of drug molecules when the pore size of MOFs is smaller than the particle size of the drug. Electrostatic adsorption is of great significance to the nano-delivery system formed using one-pot synthesis, which is often used in the synthesis of nanomedicines for bone regeneration or bone disease treatment. Risedronate was successfully loaded into ZIF-8 using one-pot synthesis, and an encapsulation rate of 64.21 ± 2.48% was achieved (Cheng et al., 2020). Sun et al. (Sun et al., 2022) also loaded 4-chloro-N-cyclohexyl-N-(phenylmethyl)-benzamide (FPS-ZM_1_) in a Co-based MOF (ZIF-67) using one-pot synthesis. The results demonstrated that post loading of FPS-ZM_1_ with ZIF-67, the particle size changed from 386.0 to 466.3 nm, the zeta potential changed from 3.63 to 3.10 mV, and the ZIF-67 delivery platform achieved a favorable sustained release effect of FPS-ZM_1_.

### 3.3 Biomimetic mineralization

Biomimetic mineralization is a loading method similar to Co-loading, in which active molecules and MNPs are mixed. However, biomimetic mineralization is predominantly used to load biomolecules, such as nucleic acids and proteins, and relies on biomolecules as nucleation sites for MNPs crystallization (Wang J. et al., 2020). The encapsulation mechanism is forming bonds/interactions between the MNPs building blocks and loading biomolecules to facilitate nucleation. In this encapsulated state, biomolecules can be protected from harsh chemical environments, heat, and degrading enzymes, while the simultaneous delayed activity or slow release of biomolecules mediated by MNPs disintegration may occur (Zou D. et al., 2020; Ha et al., 2021). The tobacco mosaic virus was loaded onto ZIF-8 via biomimetic mineralization, and its thermal and chemical stability was significantly enhanced after encapsulation (Li et al., 2018). Li et al. (Li et al., 2019b) also successfully encapsulated pDNA into ZIF-8 via biomimetic mineralization, where pDNA was uniformly distributed within the ZIF-8 nanostructure, consequently protecting it against enzymatic degradation. In a study on biomimetic mineralization of Fe_3_O_4_ NPs (Liu L. et al., 2019), 14-mer bi-functional copolypeptide was used as a template and a ginger extract was applied as an antioxidant and a size-conditioning agent. Under the cooperative effect of the peptide and ginger extract, the size and dispersibility of Fe_3_O_4_ crystals were effectively controlled. Tong et al. (Tong et al., 2015) loaded TiO_2_ NPs onto C_3_N_4_ nanosheets via the arginine-enabled biomimetic mineralization, and made Ag NPs nucleate and grow on the surface of TiO_2_ NPs.

## 4 Application scenarios of MNPs in bone diseases

### 4.1 Bone regeneration

**FIGURE 4:**
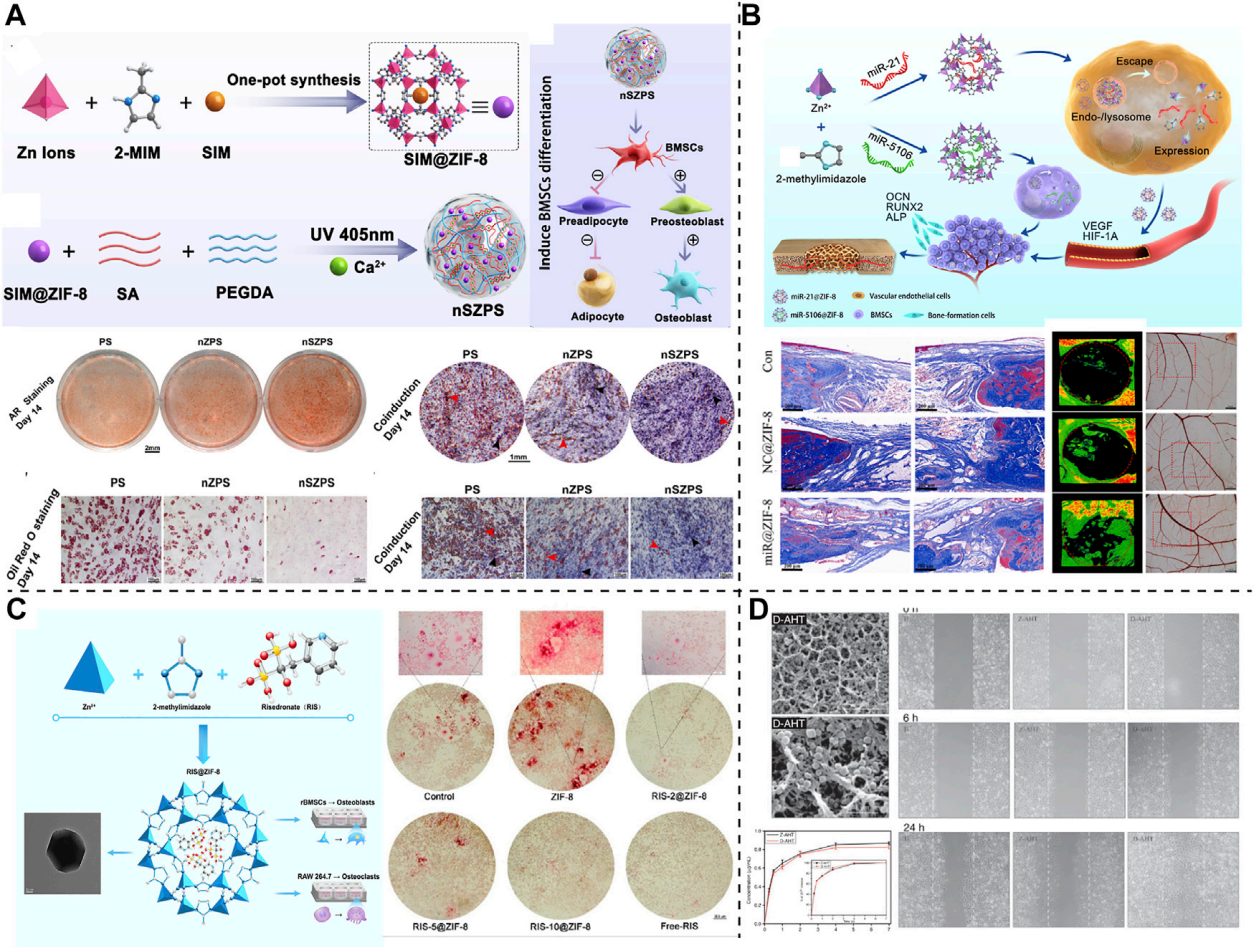
ZIF-8-based MNPs were demonstrated for building bone tissue engineering materials. **(A)** SIM@ZIF-8 carried hydrogel designed for regulating the balance of osteogenic and adipogenic differentiation, copyright 2022 Elsevier. **(B)** A ZIF-8 loading miRNA promoting vascularized bone formation, copyright 2021 Elsevier. **(C)** A ZIF-8-based MNP aiming at preventing osteoclasts, copyright 2019 ACS Publications. **(D)** Bone implants modified with DMOG@ZIF-8, copyright 2019 SAGE Publications.

The regenerative treatment of large-area bone defects imposes strict requirements on tissue engineering materials, particularly in the conditions accompanying basic diseases, such as osteoporosis, hyperlipidemia, diabetes, infection, and vascular necrosis, and the performance of the tissue engineering materials is crucial. In these challenging bone-repair scenarios, MNPs, particularly the Zn-based NPs, exhibit satisfactory application potential and are widely used for enhancing or modifying bone tissue engineering materials. For example, MNPs comprising ZIF-8 with Zn as the coordination core have assisted numerous novel bone tissue engineering materials in the completion of complex bone repair tasks (Figure 4). In the face of bone metabolism disorders and hyperlipidemia, Qiao et al. (Qiao M. et al., 2022) used ZIF-8 to encapsulate the small-molecule drug, simvastatin, to reduce serum cholesterol levels to form SIM@ZIF-8 nanocarrier particles, and SIM@ZIF-8 for enhancing the performance of poly (ethylene glycol) diacrylate (PEGDA) and sodium alginate (SA) to form SIM@ZIF-8/PEGDA/SA (nSZPS) composite biogels. The corresponding *in vivo* and *in vitro* experiments confirmed that using SIM@ZIF-8, nSZPS can inhibit adipogenic differentiation and promote osteogenic differentiation of BMSCs *in vitro*, as well as promote blood lipid lowering and osseointegration of bone in hyperlipidemia rats (Figure 3). For treating bone defects under ischemic conditions, miR-21 or DMOG, which are active components for activating blood vessels, and can also be loaded using ZIF-8, which protect and intactly deliver drugs or nucleic acids into cells, thereby completing the activation of the blood supply to the damaged bone tissue area (Zhang et al., 2019; Feng et al., 2022). In addition, for treating osteoporosis and other bone immune behavior disorders, ZIF-8 can deliver bisphosphonates (BP) to combat excessive osteoclast behavior (Cheng et al., 2020). In addition, metals, such as Ti, which are consistent with bone implants are commonly used for designing MNPs, for the design of bone tissue engineering materials (Chen et al., 2017). Saha et al. (Saha et al., 2021) used curcumin-loaded TiO_2_ nanotubes to modify Ti6Al4V implant surfaces and obtained a novel bone implant with both drug release and support properties. This Ti-based MNP-optimized implant can significantly inhibit *Escherichia coli* and *Staphylococcus aureus*, as well as effectively promote the cell adhesion, proliferation, and osteogenic differentiation of mesenchymal stem cells, which is beneficial for solving infection-related problems of Ti bone implant and its surrounding. Recently, Xue et al. (Xue et al., 2022) endowed 3D-printed polycaprolactone (PCL) scaffolds with excellent photothermal properties by loading dexamethasone sodium phosphate on CuS nanoparticle. This design can controllably release drugs under exposure to 1,064 nm NIR light irradiation and effectively promote the osteogenic differentiation of BMSCs.

Notably, the high loading capacity and stable delivery ability are not the only reasons why MNPs are preferred as bone tissue engineering modification materials. The drug release or controlled release capability of MNPs facilitates the possibility for multi-level, multi-stage, and multi-purpose bone regeneration and repair. Moreover, the metal elements in MNPs influence the physiological activities of bones. First, few metal elements (such as Zn) directly accelerate osteogenic differentiation (Table 2). For example, Liu et al. (2020c) used nano-ZIF-8 directly as a catechol–chitosan enhancer to prepare a multifunctional bone adhesive hydrogel, and the sustained-release Zn ions up-regulated the production and secretion of alkaline phosphatase, collagen1 and osteocalcin promoted the osteogenic differentiation of BMSCs and accelerated bone remodeling. Similar applications have also been observed in a few 3D-printed bone scaffolds or implants (Zhong et al., 2020). Similarly, the nano-ZnO particles also have a direct role in promoting bone repair. Studies have demonstrated that the osteogenic effect of these Zn-based nanomaterials may be achieved via upregulating the Wnt/β-catenin pathway (Gao et al., 2021). In addition to direct osteogenesis, accelerate vascularized bone repair has been achieved using NPs derived from Fe, Cu, and Mg (Zhu et al., 2020), thereby providing advantages for their constructed MNPs for bone repair. In addition, the potential hindrance of the osteoclast process is also of interest to researchers. Bai et al. (Bai et al., 2020) studied the effect of Au NPs without any drug loading on osteoclasts and observed that Au NPs can interact with the V0 domain of vacuolar-type H + -ATPase, consequently preventing its recruitment of the V1 domain, impairing osteoclast acid secretion, and inhibiting the proteolytic enzyme secretion by osteoclasts to degrade bone matrix. The inhibitory effect of Au NPs on bone resorption was demonstrated in a lipopolysaccharide-induced bone erosion mouse model. Therefore, the active regulation of osteogenesis-related processes using metal elements significantly facilitates the application of MNPs in the field of bone repair.

**TABLE 2:** Application of one Zn-based MOF (ZIF-8) in treating bone disease and regeneration.

Cargo loading	Study model	Material form	Functions	Applications	Citations
SIM	*In vitro*/*In vivo*	Biohydrogel	Inhibited adipogenic differentiation, promoted osteogenic differentiation	Bone regeneration	Qiao et al. (2022b)
MiR-21	*In vitro*/*In vivo*	Nanocomposites	Improved the osteogenic differentiation, promoted angiogenesis	Bone regeneration	Feng et al. (2022)
DMOG	*In vitro*	Coating on implants	Effect osteogenic and angiogenic activity	Bone regeneration	Zhang et al. (2019)
BP	*In vitro*	Nanocomposites	Enhanced osteogenic and antiresorptive properties	Bone regeneration, Osteoporosis	Cheng et al. (2020)
—	*In vitro*/*In vivo*	Bone adhesive hydrogel	Promoted the osteogenesis of hydrogel, inhibited bacterial activities	Bone regeneration	Liu et al. (2020d)
—	*In vitro*/*In vivo*	3D-printed bone scaffolds	Inhibited bacterial activities, promoted osteogenesis	Bone regeneration	Zhong et al. (2020)
DOX	*In vitro*/*In vivo*	Nanocomposites	Possessed stronger anticancer capability	Osteosarcoma	Xu et al. (2020)
Ald and DOX	*In vitro*/*In vivo*	Nanocomposites	Enhanced killing tumor cells of bone metastases	Bone metastasis	Xue et al. (2020)
Cu_2_-_X_Se	*In vitro*/*In vivo*	Nanocomposites	Suppressed the tumor cells and reduced the erosion of bone tissue	Bone tumor	Zou et al. (2022)

### 4.2 Osteomyelitis

Osteomyelitis is an inflammatory/infectious bone disease and considerably affects the daily life of patients, and it is commonly occurring in people that are older, diabetic, or with poor general health. Osteomyelitis is often caused by pyogenic bacteria, such as *Staphylococcus aureus* and *Staphylococcus* epidermidis, and fungal infections, such as *Haemophilus* influenzae and *Brucella* suis fungi. It may also be a complication of orthopedic surgery, tooth extraction, or facial plastic surgery. The predominant treatment approach currently employed is the administration of systemic antibiotics, which is less effective at the localized infection site. The topical use of MNPs encapsulated with antibiotics or MNPs with targeted functions is favorable choice to enhance the therapy of osteomyelitis, delay drug action time, reduce the systemic side effects, and reduce osteonecrosis. Abdulrehman et al. (Abdulrehman et al., 2020) synthesized Ag–Cu–boron (ACB) alloy NPs and obtained ACB–OBAb nano-delivery system via the Cadherin-11 antibody (OBAb) coupling. It has been demonstrated that ACB–OBAb can target osteoblasts and effectively inhibit *Staphylococcus aureus* inside and outside the infected osteoblasts. This study demonstrates the significance of MNPs in reducing systemic toxicity and targeting bone infection and inflammation. Escobar et al. (Escobar et al., 2019) used Ti dioxide film (MTF) loaded with gentamicin and conjugated human recombinant BMP-2 on its surface. This delivery platform can effectively inhibit the colonization of *Staphylococcus aureus* as well as significantly enhance the MC3T3-E1 preosteoblastic cell attachment, proliferation, and differentiation. Liu et al. (Liu et al., 2020b) used the Au NPs synthesized using *Acetobacter* and Gluconobacter to load ginsenoside compound K (CK) and surface-conjugated CopA3 to obtain the GNP–CK–CopA3 nano-delivery system. GNP–CK–CopA3 improved the LPS-induced production of nitric oxide (NO) and ROS and inhibited the mRNA and protein expressions of pro-inflammatory cytokines in macrophages, combined with significant anti-inflammatory effects.

In addition to the treatment of osteomyelitis via high-efficiency drug loading, anti-inflammatory or antibacterial activities are attributes of most MNPs that can release metal ions, making them effective candidates for the topical treatment of osteomyelitis. The powerful antibacterial, antifungal, and osteoinductive properties of Ag-based nano-delivery platforms have been extensively validated and are exceedingly the most commonly used commercial NPs (Hauser and Nowack, 2021). In addition, there is significant evidence that Cu-based, Ti-based, Zn-based, Palladium-based, iron-based, Co-based and other nanomaterials exhibit favorable bactericidal properties (Rojas-Andrade et al., 2017). Additionally, compared with traditional antibiotics, MNPs avoids the limitations caused by drug resistance. Marsich et al. (Marsich et al., 2013) prepared alginate/hydroxyapatite composite scaffolds containing Ag NPs and demonstrated that the presence of Ag NPs endowed the scaffolds with favorable sterilization against both Gram+ and Gram− bacterial strains and did not affect the ability of the scaffold to promote the proliferation of osteoblasts. Arenas-Vivo et al. (Arenas-Vivo et al., 2019) formed nanocomposites with MIL-125(Ti)NH2 composite Ag NPs. This nanocomposite exhibits a favorable effect against *Staphylococcus aureus* biofilm under the triple action of the intrinsic bactericidal activity of MIL-125(Ti), bactericidal properties of Ag NPs, and photoactivity of UVA irradiation. This study provides a substantial basis for MNPs to inhibit bone implant biofilm formation and treat osteomyelitis caused by bacterial infection. According to reports (Krumdieck et al., 2019), TiO_2_ can be used as an effective antibacterial coating material owing to its unique photocatalytic properties, which can effectively reduce *E. coli*. In a study, magnetic Co. ferrite NPs were demonstrated to exhibit inhibitory effects on Gram-negative *Escherichia coli* and Gram-positive *Staphylococcus aureus*, *Candida* parapsilosis, and *Candida* albicans. Moreover, the antibacterial effect was improved with increasing the Co2+ content (Zalneravicius et al., 2018). The cooperation of different metal NPs sometimes brings new ideas for the treatment of deep osteomyelitis. Fe_3_O_4_ NPs and Au NPs were combined to act as the nuclear component of an engineered macrophage with a nuclear–membrane structure. The nuclear component can produce abundant ROS and heat under microwave irradiation thus suppressing inflammatory responses, killing bacteria *in situ*, simultaneously promoting osteoblast differentiation for osteomyelitis (Fu et al., 2021).

### 4.3 Bone tumor

There are several commonly occurring types of bone tumors, such as osteosarcoma and bone metastases originating from breast or prostate cancer. The common symptoms of bone tumors are severe pain, pathological fractures, bone marrow aplasia, and hypercalcemia, which induce considerable pain in patients. CT, radiotherapy, and ablation are the commonly used non-surgical methods for treating bone tumors. DOX, cisplatin, ifosfamide, BP, tetracycline, denosumab, cabozantinib, mesna, methotrexate, etc. Are commonly used as CT drugs for bone tumors. To reach bone tissues via systemic administration, drugs require to penetrate the blood–bone marrow barrier including the clefts of bone marrow sinusoidal capillaries with diameters of 80–100 nm (Sarin, 2010). Therefore, MNPs are favored by bone tumor specialists because of their small sizes and high loading capacities. Fang et al. (Fang et al., 2020) synthesized a nanoscale Co–ferrocene MOF (Co–Fc NMOF) and loaded glucose oxidase with this nanoplatform to construct Co–Fc@GOx, which plays the role of a cascade enzymatic/Fenton catalytic platform to cure cancer via CDT. In addition, a few scholars (Raghubir et al., 2020) delivered riluzole through two different shapes of iron oxide NPs (nanocages or nanospheres, 15 ± 2.5 nm), and significantly induced tumor tissue apoptosis in the osteosarcoma model mice. Mokhtari et al. (Nezhad-Mokhtari et al., 2019) loaded methotrexate into the empty face-centered cubic lattice and two-dimensional tunnels of Cu-MOF to form nano-delivery ions (Cu-MOF/MTX), and further inserted Cu-MOF/MTX into a series of synthesis experiments of novel microspheres (Cu-MOF/MTX@GM) from pH-sensitive gelatin microsphere biopolymers, demonstrating that the microspheres are suitable for targeted anticancer drug delivery. In a report (Sha et al., 2021), metal manganese (Mn) was *in situ* doped into Au core mesoporous silica NPs to construct a multifunctional MNPs (Au@MMSN). Au@MMSN was further loaded with Ald and DOX (DOX@Au@MMSN-Ald) for osteosarcoma treatment. It was confirmed that the Au and Mn ions released from the nanocomposite could be used for computer tomography and MR dual-modality imaging for *in vivo* localization. At the same time, DOX@Au@MMSN-Ald can release antitumor drugs responsively to realize the combined treatment of CT and CDT for osteosarcoma.

MNPs can load drugs for bone tumor CTP and show considerable potential in PTT, PDT, sonodynamic therapy, and other new noninvasive solid tumor therapy or multimode combined therapy. Recently, Geng et al. (Geng et al., 2021) reported ultrafine W-doped TiO_2_ (W- TiO_2_) nanorods for sonodynamic−chemodynamic combination tumor therapy. They observed that W doping narrows the band gap from 3.2 to 2.3 eV, enhancing the TiO_2_ acoustodynamic properties; W5+ doping endows W- TiO_2_ nanorods with Fenton-like reactivity; W6+ doping promotes the transfer of endogenous transformation of glutathione to W5+ ions, thereby enhancing the chemical kinetic activity of W- TiO_2_ and changing the tumor microenvironment. *In vivo* experiments demonstrated that W- TiO_2_ enhances tumor eradication in an osteosarcoma model under single ultrasound irradiation. Cheng et al. (Cheng et al., 2021) used a mixed-metal Cu/Zn-MOF for integrating Mn2+ and MnO2 and loaded the photosensitizer ICG to form nanocomposites. The contained ICG can realize photothermal imaging and PTT under laser irradiation. The nanocomposite exhibits fluorescence imaging and PDT capacity by releasing ICG upon reaching the tumor site and can produce cytotoxic OH by releasing Cu+/Mn2+ and scavenging glutathione to exert CDT effect. The study demonstrates that MNPs are excellent candidate materials for PTT/PDT/CDT of bone tumors. Recently, Zou et al. (Zou et al., 2022) developed a composite nanoplatform using ZIF-8–capped Cu_2_-_X_Se and used it for CDT and PTT of bone tumors. Cu_2_-_X_Se was released with the cleavage of ZIF-8 under acidic microenvironment in tumor and subsequently degrade into Cu^+^ and Cu^2+^ to initiate a Fenton-like reaction inducing CDT. At the same time, Cu_2_-_X_Se can also induce PTT effect and inhibit tumor cells and osteoclasts. This ZIF8-capped nanomedicine effectively demonstrates the potential value of MNPs for advanced therapy in bone tumors.

### 4.4 Osteoarthritis

Osteoarthritis is a chronic disease characterized by the progressive degeneration of articular cartilage, abnormal reduction of joint lubrication, and synovial inflammation. Joint pain as well as joint damage and dysfunction are the primary clinical symptoms of osteoarthritis (Litwic et al., 2013). Several common drugs, including anti-rheumatic drugs, non-steroidal anti-inflammatory drugs, glucocorticoids, and newly discovered biological agents, are used to treat osteoarthritis (Mackenzie and MacDonald, 2010). Although intra-articular drug injection is a characteristic administration mode for treating osteoarthritis, the free drug is rapidly cleared from the joint cavity, leading to increased complications and decreased drug bioavailability (Kompel et al., 2019). Based on this, MNPs have been extensively explored as a drug-loading vehicle for osteoarthritis (Table 3). Xiong et al. (Xiong et al., 2020) developed a pH-responsive Fe-based MOF system MOF@HA@PCA loaded with an anti-inflammatory protocatechuic acid (PCA) and modified with hyaluronic acid (HA), which can respond to the acidic microenvironment and gradually released for treating osteoarthritis PCA, which downregulates the expression of osteoarthritis inflammatory markers and promotes the expression of cartilage-specific markers, significantly reducing IL-1β-induced synovial inflammation in both joints and chondrocytes. Li et al. (Li et al., 2020b) generated five poly (amidoamine) dendrimer-entrapped Au NPs (Au DENPs) that simultaneously delivered antioxidant α-TOS and anti-inflammatory antiTNF-α siRNA (Au DENPs/TNF-α siRNA complex thing). The complex can significantly enhance the antioxidant capacities of macrophages and down-regulate inflammatory cytokines in arthritis mouse models, thereby achieving a combined antioxidant and anti-inflammatory therapy for joint inflammation. MNPs also can contribute uniquely to stem cell therapy for osteoarthritis. The CuS@MnO2 NPs loaded with metformincan could be targeted for uptake by MSCs which further were used as stem cell therapy for osteoarthritis. MSCs modified with this MNPs were validated to exhibit an increased capability of anti-inflammation and chondrogenesis, and effectively relieve osteoarthritis symptoms (Lu et al., 2022).

**TABLE 3:** Application scenarios of MNPs in bone diseases.

Application scenarios in bone diseases	MNPs	Cargo loading		Focus points	Citations
Bone regeneration	ZIF-8	SIM		Osteogenic and adipogenic differentiation	Qiao et al. (2022)
	ZIF-8	miR-21		Vascularized Osteogenesis	Feng et al. (2022)
	TiO_2_	DMOG		Osteogenic differentiation	Zhang et al. (2019)
	ZIF-8	Bisphosphonates		Osteoclast control	Cheng et al. (2020)
	TiO_2_	Curcumin		Antibacterial	Saha et al. (2021)
	CuS	Dexamethasone sodium phosphate		NIR response	Xue et al. (2022)
	ZIF-8	—		Bone remodeling	Liu et al. (2020c) Zhong et al. (2020)
	ZnO	—		Osteogenic differentiation	Gao et al. (2021)
	Au	—		Inhibitory effect on bone resorption	Bai et al. (2020)
Osteomyelitis	Ag−Cu−B	Cadherin-11 antibody		Inhibition of bone inflammation	Abdulrehman et al. (2020)
	TiO_2_	Gentamicin/BMP-2		Antibacterial,	Escobar et al. (2019)
	Au	GNP−CK−CopA3		Anti-inflammatory effects	Liu et al. (2020b)
	Ag	—		Antibacterial	Hauser and Nowack (2021) Marsich et al. (2013)
	MIL-125(Ti)NH_2_ composite Ag	—		Inhibition of bone implant biofilm formation	Arenas-Vivo et al. (2019)
	TiO2	—		Antibacterial	Krumdieck et al. (2019)
	Magnetic Co ferrite NPs	—		Antibacterial	Zalneravicius et al. (2018)
	Fe_3_O_4_ NPs and Au NPs	—		Microwave response and suppressing inflammatory	Fu et al. (2021)
Bone tumor	Co-ferrocene MOF	Glucose oxidase		CDT	Fang et al. (2020)
	Iron oxide	Riluzole		Tumor apoptosis	Raghubir et al. (2020)
	Cu-MOF	Methotrexate		Targeted drug delivery	Nezhad-M et al. (2019)
	Mn - Au - mesoporous silica NPs	Ald and DOX		CT and CDT	Sha et al. (2021)
	TiO_2_	W		Sonodynamic − chemodynamic combination tumor therapy	Geng et al. (2021)
	Cu/Zn-MOF	ICG		PTT, PDT, and CDT	Cheng et al. (2021)
	ZIF-8	Cu2-XSe		CDT and PTT	Zou et al. (2022)
Osteoarthritis	Fe-MOF	PCA		Anti-osteoarthritis	Xiong et al. (2020)
	Au	α-TOS and anti-TNF-α siRNA		Combination of antioxidant and anti-inflammatory	Li et al. (2020)
	CuS@MnO_2_	Metformincan		Anti-inflammation and chondrogenesis	Lu et al. (2022)
	Au-coated CuS	Vasoactive intestinal peptide and HA		Removing hyperplasia	Huang et al. (2021)
	Au-coated Fe_3_O_4_	—		Inhibiting joint edema and inflammation	Carneiro et al. (2020)

Similarly, MNPs provide new avenues for osteoarthritis treatment owing to their excellent light responsiveness, strong plasticity, and self-anti-inflammatory properties. Huang et al. (Huang et al., 2021) reported a metal/semiconductor composite (Au NR@CuS) comprising octahedral Cu sulfide shell and Au nanorod core, loaded with vasoactive intestinal peptide and HA to form VIP–HA–Au NR@CuS NPs. It was experimentally demonstrated that the nanocomposite generated a photothermal effect under laser irradiation and introduced additional OH for PDT under the integration of a Fenton-like reaction and Au NR and CuS semiconductor photocatalysts; targeting synovial cells under the action of active intestinal peptides and HA, thereby exerting the combined effect of PTT, PDT, and CT, significantly removing the hyperplastic synovium, reducing joint inflammation symptoms. In addition, it has been reported (Carneiro et al., 2020) that Au-coated superparamagnetic iron oxide NPs could more significantly reduce the number of immunostaining positive cells of TNF-α and IL-1β in synovium compared with the drug methotrexate alone, thereby inhibiting joint edema and inflammation.

## 5 Challenges of MNPs

Although the applications of MNPs in bone tissue engineering and the management of various bone-related diseases have been well reported, only a few MNPs have entered the market evaluation stage and received final approvals. Numerous challenges remain to be overcome in the translation of MNPs from basic experiments to clinical applications in orthopedics, which may originate from multiple aspects and fields. We analyzed the reasons and summarized them into the following points:

### 5.1 Agglomeration

Several reports have mentioned the agglomeration of MNPs, which limits MNPs functionality in the nano-scale, as well as their dispersibility and stability. A few scholars believe that electrostatic interactions, which is size-dependent, cause this agglomeration (Chandrakala et al., 2022). Agglomeration *in vivo* hinders the dispersibility of MNPs, which challenges the targeting efficacy and biosecurity of MNPs. Apparently, agglomeration may also affect the *in vivo* behavior of MNPs, such as the cellular uptake, inter-tissue transport, penetration, diffusion, and biocompatibility (Sengul and Asmatulu, 2020), which hinder the application of MNPs as active molecules or therapeutic agent delivery vehicles. Surface functional modification of MNPs is currently a commonly used method to overcome the limitations of agglomeration instability and improve biological applications. The surface functionalization of MNPs can be achieved via techniques such as coordination binding of unsaturated metal sites, ligand exchange, and covalent binding with pre-functionalized linkers (Kainz and Reiser, 2014; Nolte et al., 2017). The thermal and chemical stability of MOFs can be significantly improved using amino-modified MOFs in 2-aminoterephthalic acid ligands (Chen et al., 2019). Surface PEGylation is an FDA-approved technique (Veronese and Pasut, 2005). In addition, complexation with other nanomaterials (polymer NPs, liposomes, cubes, etc.) can reduce MNP agglomeration (Phuong et al., 2019). These methods impose strict requirements on process flow, cost, and technical parameters. Moreover, all MNPs cannot overcome the agglomeration limitation via the aforementioned methods. The surface chemical properties of MNPs are significantly vary, and the chemical groups or compounds that can be matched are complex. Methods to overcome MNP agglomeration remains a topic worthy of future research.

### 5.2 Toxicity

The toxicity of MNPs has always been a hot research topic. In addition to the agglomeration phenomenon mentioned above, there are several reasons for MNP toxicity. The recognized high specific surface area of MNPs leads to high surface reactivity, which is a double-edged sword that may bring unexpected toxicity mechanisms to MNPs (Mahana et al., 2021). The small sizes of MNPs promote the cellular uptake rate, which subsequently increases bioavailability as well as enhances toxicity (Yang et al., 2013). In addition, MNPs release numerous metal ions with a bactericidal effect, but excessive metal ions can impair the electrolyte balance of the body fluid environment and the normal ion exchange of cell membranes, consequently resulting in cytotoxicity (Wang et al., 2016a). It has been reported that heavy metal ions accumulated in osteoblasts and chondrocytes can lead to cellular dysfunction by replacing essential elements in enzymes and disrupting the conformation of the active site, thereby increasing the risk of osteoporosis and osteoarthritis (Krizkova et al., 2016). Organic linkers, metal ions, solvents, and chemical residues formed during MNP synthesis also cause toxicity, which still requires attention and should be resolved. Although a few scholars (Huang et al., 2020; Ting et al., 2022) have functionally modified MNP surfaces by targeting ligands, attempting to reduce systemic toxicity through the targeting of MNPs, MNP toxicity remains a major problem that limits its clinical application.

### 5.3 Complexity

The application of MNPs in orthopedics, particularly in tissue engineering materials, often requires the cooperation of materials such as scaffolds, gels, and biofilms. The type of disease, drug resistance, implantation site, mechanical properties, and several other requirements should be comprehensively considered. This requires the precise control of MNPs by nanomedicine experts, as well as a high degree of coordination among orthopedic surgeons, biomedical scientists, and biomechanical engineers (Schuemann et al., 2020). In addition, the repair/treatment requirements of bone defects/diseases in different zones of the epiphysis, diaphysis, metaphysis, and joints are different. The most important requirement for diaphyseal healing is the establishment of mechanical strength; for the metaphysis, joints, both mechanical strength and flexibility require to be considered. Therefore, the mechanical, physiological, pathological, immune biochemical, and surgical conditions for the design and implementation of MNPs into scaffolds should be comprehensively considered. That is, for different orthopedic problems, appropriate MNPs should be specially designed for the corresponding needs. Particularly, the selection of optimal drugs or active ingredients, metal ions, organic ligands, supporting biomaterials, machines, preparation techniques is a complex interdisciplinary task.

## 6 Conclusion

The rapid development of MNPs provides new avenues for innovating the diagnosis and treatment of bone diseases and regeneration. The advantages of MNPs are significant: high loading rate, excellent antibacterial properties, functionally modified surface, tunable pore volume and size, acceptable biocompatibility, and strong mechanical properties as well as a few unique features such as magnetic properties, light responsiveness, and pH responsiveness. Various types of MNPs, such as MOFs, metal nanoplatforms, and metal oxide nanoplatforms, exist. Ag NPs and Ag_2_O NPs are exceedingly the most commonly used commercial nanoplatforms. The current mainstream research is focused on the development and application of MOFs. In addition to small-molecule drug MNPs, various therapeutic agents, such as proteins, viruses, peptides, and RNA, can be effectively delivered. Moreover, several delivery methods can be used, such as post-loading, Co-loading, and biomimetic mineralization. MNPs can be used in various orthopedic scenarios, most commonly in bone tissue engineering regeneration and can also play a favorable role in bone regeneration in a few osteoporotic diseases. In addition, MNPs have significant potential in treating diseases, such as osteomyelitis caused by various infections, bone metastases from different sources, osteosarcoma, and osteoarthritis. However, MNPs still have limitations; for instance, the problems of aggregation and toxicity require to be further overcome. In the future, multidisciplinary efforts are required to promote the clinical application of MNPs in orthopedics.
